# Clinic and electromyographic results of latissimus dorsi transfer for irreparable posterosuperior rotator cuff tears

**DOI:** 10.1186/s13018-014-0083-6

**Published:** 2014-11-08

**Authors:** Ricardo De Casas, Matías Lois, Myriam Cidoncha, Miguel Valadron

**Affiliations:** Department of Orthopedic Surgery, Clinica Traumacor, Ronda de Nelle 72, 15005 A Coruna, Spain; Department of Orthopedic Surgery, Centro Gallego de Buenos Aires, Avenida Belgrano 2199, 1094 Buenos Aires, Argentina; Department of Physical Medicine, Clinica Traumacor, Ronda de Nelle 72, 15005 A Coruna, Spain

**Keywords:** Rotator cuff tear, Irreparable, Latissimus dorsi transfer, Surface electromyography

## Abstract

**Background:**

This study examines the clinical and electromyographic results of latissimus dorsi transfer (LDT) using a combined open and arthroscopic technique for the treatment of symptomatic irreparable posterosuperior rotator cuff tears.

**Methods:**

Between 2006 and 2009, LDT was performed in 14 patients (mean age 59 years) with massive and symptomatic irreparable posterosuperior rotator cuff tear. The patients were examined preoperatively and postoperatively with mean follow-up of 52 months using the Constant score, and the integrity of the latissimus dorsi (LD) transfer was assessed by ultrasound in all cases and by MRI in ten cases. The functional activity of the LD transfer was compared to the non-operated side using surface electromyography.

**Results:**

All patients demonstrated a significant improvement in the Constant score (*p* = 0.001), from a preoperative score of 33 points (range 10–55 points) to a postoperative score of 59 points (range 13–80 points). The subjective assessment score was good to excellent in 12 patients (85%), and 11 patients (78%) would be willing to undergo surgery again. Integrity of the transferred tissue was confirmed in 13 of the 14 cases using ultrasound and MRI. Surface electromyographic signal showed increased activation of the transferred latissimus dorsi when performing active movements of external rotation (*p* = 0.002) and abduction-elevation (*p* = 0.009).

**Conclusions:**

Our results indicate that LDT significantly improves function and diminishes pain in patients with a massive posterosuperior rotator cuff tear. The combined open and arthroscopic technique preserves the deltoid muscle and controls the LD tendon reinsertion. Surface electromyographic signal confirms the active function of the transferred muscle.

**Electronic supplementary material:**

The online version of this article (doi:10.1186/s13018-014-0083-6) contains supplementary material, which is available to authorized users.

## Background

Massive symptomatic posterosuperior rotator cuff tear is defined as a tear with a diameter of more than 5 cm that affects the supraspinatus and infraspinatus tendons [1] and presents a complex and controversial therapeutic problem for the orthopedic surgeon.

Different surgical techniques have been described in the cases of irreparable lesions or repair failures, including the deltoid flap, arthroscopic debridement, biceps tenotomy-tenodesis, tuberoplasty, and use of allografts and synthetic mesh [2-5]; however, the results have been limited and unpredictable. In elderly patients, the use of a reverse total shoulder arthroplasty, associated with latissimus dorsi transfer (LDT), has shown good results, although there are still questions about the durability of the transfer and salvage procedure options in the case of failure [6].

LDT was originally introduced by Gerber et al. in 1988 [7] to repair a posterosuperior cuff defect and to restore external rotation mobility. Since then, LDT has been performed as a primary surgery and also for cuff repair failure, achieving promising results in functional improvement [8-11]. Several investigations of the LDT using surface electromyography (EMG) have reported increased activity of the original muscle in its new function [8,9,12,13].

This paper presents a retrospective study of a series of active patients with irreparable posterosuperior rotator cuff tears that were treated with LDT using a combined open and arthroscopic technique. Our purposes are to analyze the clinical and functional outcomes and the varied reported surgical techniques and to evaluate the activity of the transferred latissimus dorsi (LD) muscle using surface EMG.

## Methods

Between 2006 and 2009, 14 patients (10 males and 4 females) with massive and symptomatic irreparable posterosuperior rotator cuff tear underwent LDT by a single surgeon. Prior to surgery, all patients provided informed written consent. This research was carried in compliance with the Helsinki Declaration, and the Ethical Committee of the Traumacor Clinical gave the approval. The diagnosis was made by clinical examination, ultrasound (Esaote, Mylab 25), and MRI (Esaote, Opera). All patients showed weakness of active abduction-elevation and external rotation and had a complete tear of the supra and infraspinatus tendons. The study group included seven manual workers and five cases were failures of a previous rotator cuff repair, including three related to working conditions. There was neither deltoid muscle nor axillary nerve lesions. Surgery was indicated in the presence of significant levels of pain and dysfunction, non-responsive to oral medications, and physical therapy. Massive irreparable tears were defined as those with grade 3 Patte tendon retraction and grade >2 muscular atrophy, according to Thomazeau. Exclusion criteria were glenohumeral arthritis (Samilson grade >1) and superior humeral migration with less than 5 mm acromiohumeral distance.

All patients were examined preoperatively and postoperatively using the Constant score by an independent observer. The integrity of the LD transfer was assessed by ultrasound in all cases and by MRI in ten cases performed by an independent radiologist.

**Figure 1:**
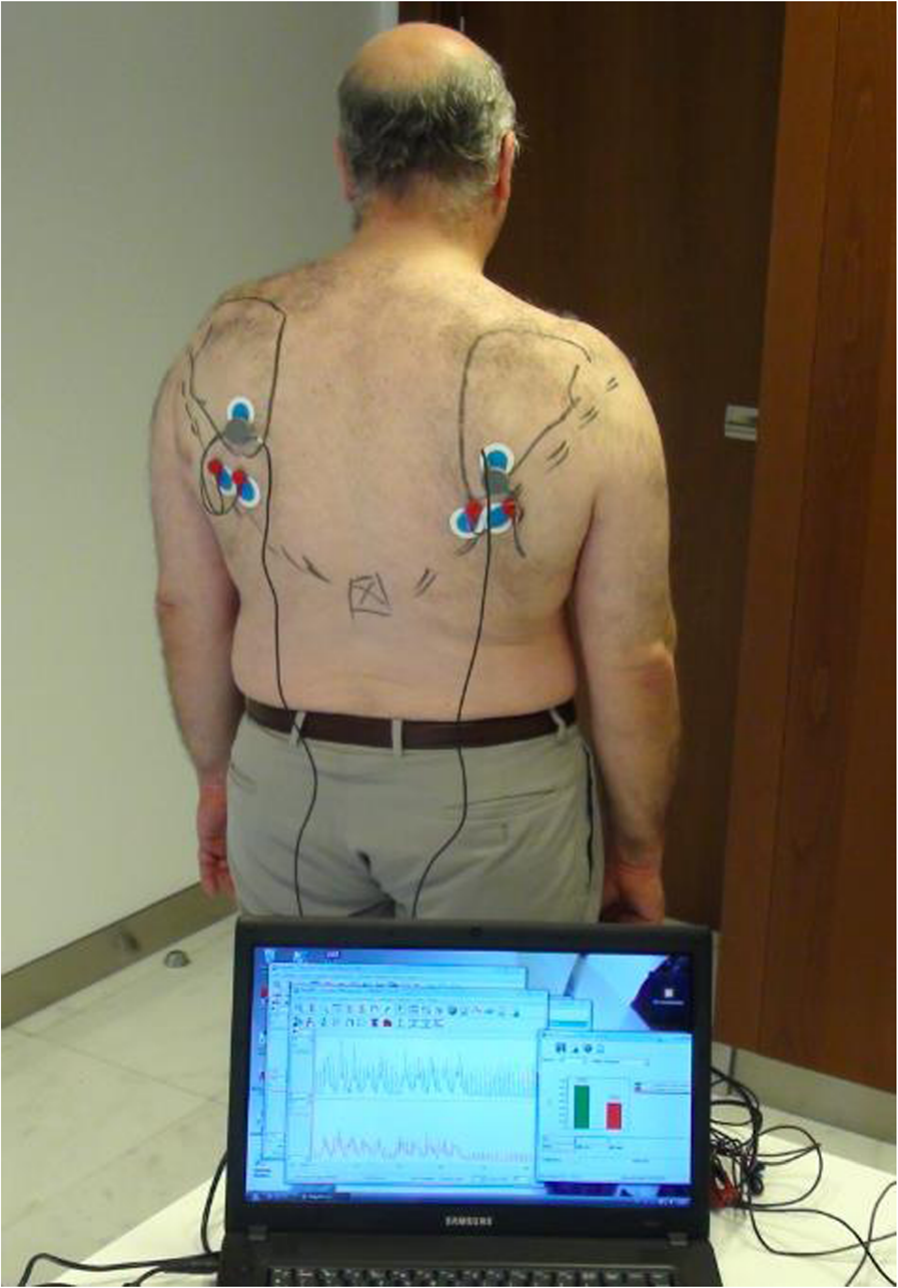
**Surface electromyographic study and position of electrodes.**

The activity of the LD transfer was compared to the non-operated side using surface electromyography (8-channel EMG Megawin 6000, Mega Electronics Ltd.). With the patients in standing position, bipolar electrodes were positioned in line with the muscle fibers, from L1 vertebra to posterior axillary crease and two channels (right and left) were used for simultaneous registration of both operated and non-operated side (Figure 1). Patients were instructed to perform separately three sets of five movements of external rotation with the arm at the side and the same for combined 90° abduction-elevation in scapular plane. For each type of movement, the activity level of the operated side was compared as a percentage of the non-operated side defined as 100% (see Additional file 1).

### Surgical technique

Surgical technique was performed by a senior author (RdC) and was modeled on the techniques reported by Habermeyer et al. [9] and Herzberg et al. [14]. The procedure was performed at a single time in the three phases, with the patient in the lateral position (see Additional file 2).

#### Phase 1: standard arthroscopy

- Joint surface exam so as to confirm the viability of the LD transfer.
- Assessment of the long head of the biceps tendon: absent in six cases and tenotomy was performed in the eight remaining cases.
- Assessment of the subscapularis tendon: two partial and one total rupture, which were repaired with suture anchors.
- Insertion of two to three intraosseous suture anchors at the level of the posterosuperior region of the greater tuberosity, for reinsertion of the LD tendon.

#### Phase 2: open surgery

We used a posterior axillary approach, making a 10–12-cm incision in line with the lateral border of the LD muscle. The teres major and LD tendons were dissected to their insertion sites on the medial lip of the bicipital groove. During dissection of the LD, the arm was internally rotated to facilitate exposure of the tendon insertion. At this level, the radial nerve crosses the LD in an anterior-inferior position, and the circumflex vessels and axillary nerve are visualized immediately proximal.

**Figure 2:**
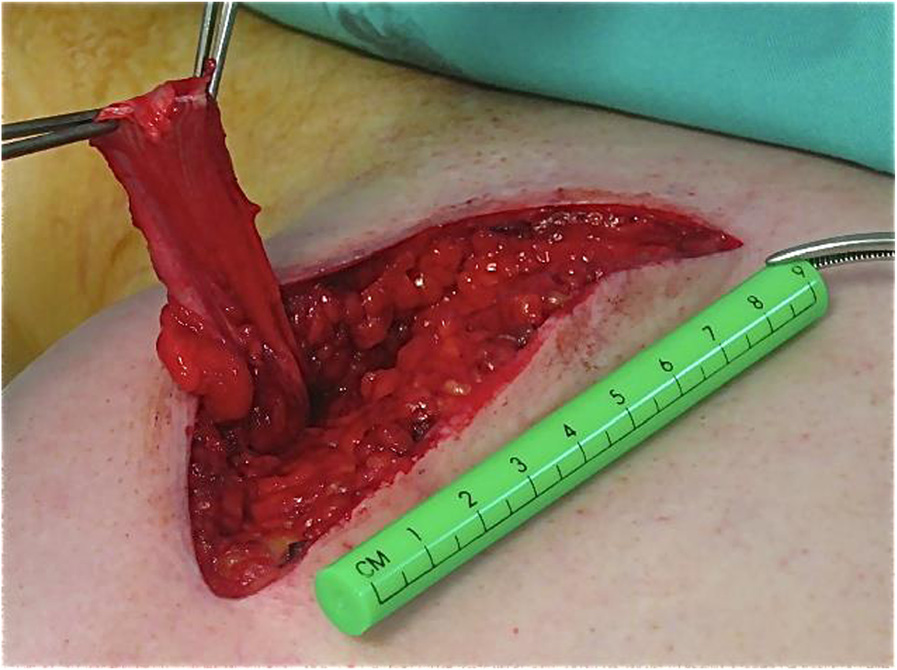
**Posterior approach and released latissimus dorsi tendon.**

Once the LD tendon was detached, we proceeded to release the muscle unit subcutaneously to achieve a satisfactory length (Figure 2). We also dissected the thoracodorsal neurovascular pedicle to confirm that there was no tension when reinserting the transfer onto the greater tuberosity.

#### Phase 3: LDT reinsertion

**Figure 3:**
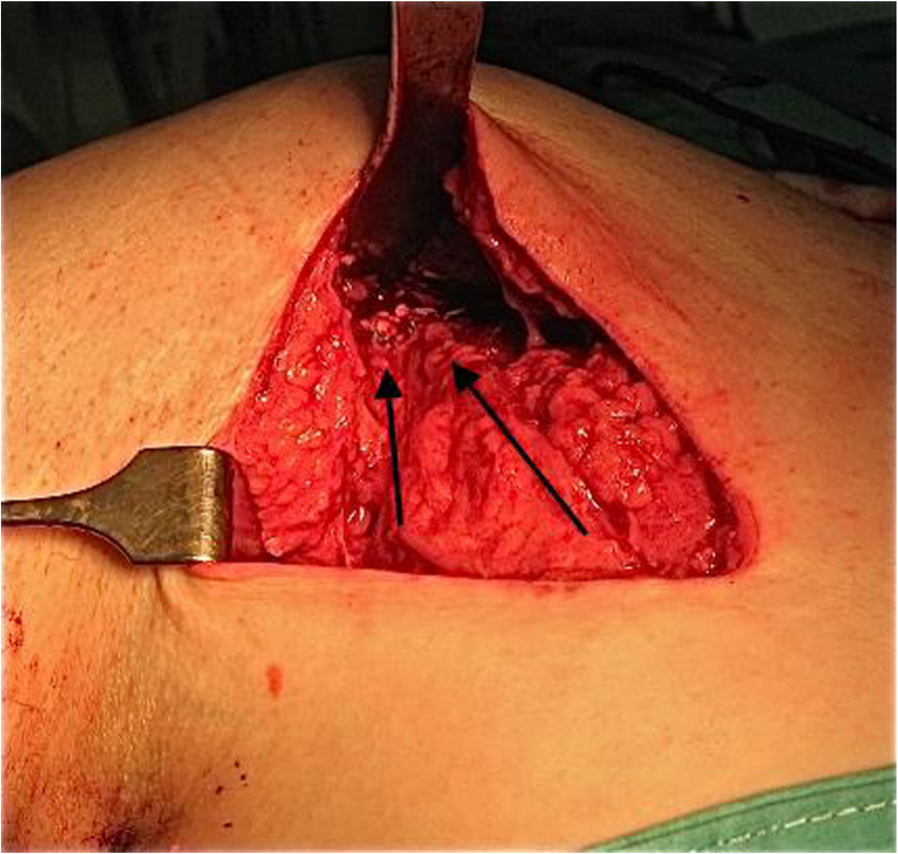
**Latissimus dorsi tendon reattached to greater tuberosity (** ***arrows*** **show the direction of the tendon).**

A subdeltoid tunnel was developed by blunt dissection between the teres minor and the deep surface of the deltoid. Next, we moved the shoulder in abduction and external rotation for exposure of the posterosuperior part of the greater tuberosity. In 11 of the patients, the access to the greater tuberosity was easily feasible and, by retrieving the sutures of the anchors through the posterior approach, the LD tendon was fixed to the previously placed suture anchors while maintaining the shoulder in abduction-external rotation (Figure 3). In the remaining three patients, the greater tuberosity was not easily accessible for a direct tendon reinsertion and we proceeded to an arthroscopic technique. One limb of each of the double suture threaded anchors was retrieved through the posterior approach and sutured to the LD tendon in a Masson-Allen way. Next, we placed a traction suture in the LD tendon, and, using a suture retriever, it was passed to an anterolateral portal. Finally, all anchor sutures were transferred to a working lateral portal, and the transferred tendon was secured to the suture anchors under arthroscopic control.

### Postoperative treatment

After surgery, we placed a rigid orthosis for 5 weeks to keep the shoulder at 30° of abduction with neutral rotation. During this time, the shoulder was passively mobilized to 90° of elevation and abduction, avoiding internal rotation. Active mobilization exercises were started in the sixth week, and free use of the shoulder was allowed in the eighth week.

From the eighth week onwards, we instructed the patients to maintain active LD contraction during elevation and external rotation movements. Using biofeedback from surface electromyography and through visualization of LD activity, the patients learned how to initiate and perform abduction-elevation and outward rotational movements of the arm.

### Statistical analysis

Statistical comparison between the nonparametric pre- and postoperative data was performed using the Wilcoxon signed-rank test, with a significance level of *p* <0.05. The software SPSS 19.0 for Windows was used for statistical analysis.

## Results

The 14 patients were followed up over mean interval of 52 months (range 36–77 months) after LDT, and the average patient age was 59 years (range 52–66 years). Nine shoulders were on the dominant side. The arthroscopic findings included two partial and one total ruptures of the subscapularis tendon which were repaired with suture anchors; the absence of the long biceps in six cases and biceps tenotomy was performed in the remaining eight cases.

**Figure 4:**
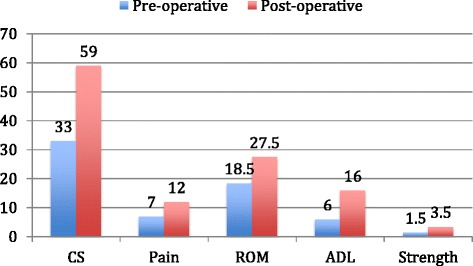
**Comparative preoperative and postoperative Constant score (CS) and its parameters.**

The mean Constant score increased from 33 points preoperatively (range 10–55 points) to 59 points (range 13–80 points) postoperatively, an increase of 26 points (*p* = 0.001). The patients had significant improvement in all parameters including pain, activities of daily living, and active mobility; although the patients had less improvement in abduction strength (from 1.5 points preoperatively to 3.5 points postoperatively) (Figure 4).

The mean preoperative score for pain was 7 points, and the pain score increased to 12 points (*p* = 0.006) after surgery. The mean score for the activities of daily living increased by 10 points (*p* = 0.001), from 6 to 16 points.

In terms of active mobility, shoulders showed a significant improvement in flexion, abduction, and external rotation. On average, they gained 48° of flexion (*p* = 0.003; from 84° to 132°), 45° of abduction (*p* = 0.003; from 80° to 125°), and 16° of external rotation (*p* = 0.03; from 12° to 30°) after the surgery.

In particular, three of the four cases of pseudoparalysis (preoperative active abduction-elevation less than 40°) showed an overall improvement in shoulder mobility. The fourth patient had a complete tear of the subscapularis tendon that was repaired in the same surgical procedure, but that patient only regained a small degree of external rotation.

Of the 14 patients, 10 (71%) were very satisfied, 2 (14%) were moderately satisfied, and 2 (14%) were dissatisfied with the surgery. A total of 11 patients (78%) said that they would be willing to undergo surgery again.

Ultrasound and MRI confirmed transfer integrity in 13 cases and identified late postoperative detachment in 1 case, which was not revised.

**Figure 5:**
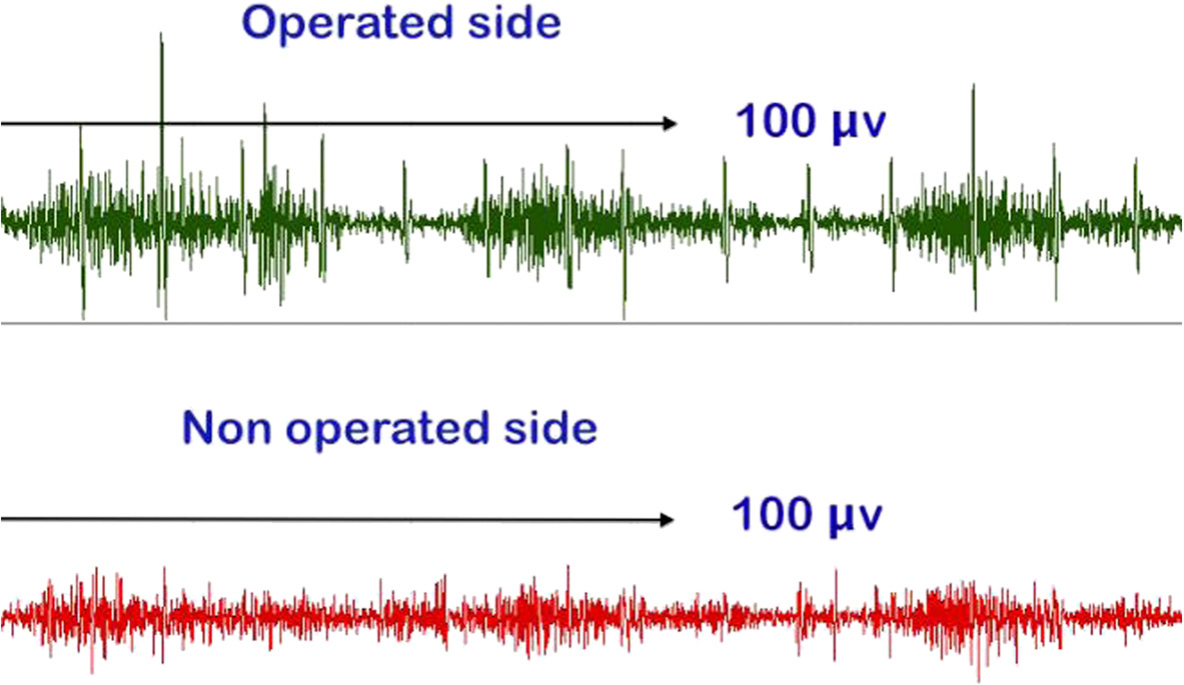
**Electromyography showing a higher activity for external rotation in the operated side than in the non-operated side.**

**Figure 6:**
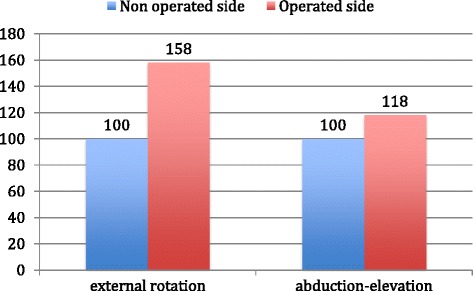
**Comparative median value of surface electromyographic activity of latissimus dorsi between non-operated and operated side.**

Surface electromyography of the LDT was recorded in 12 of the 13 patients, with anatomic integrity of the transferred muscle. Compared to the non-operative side, we observed a higher level of activity when performing either external rotation (Figure 5) or combined abduction-elevation in the LDT side. With respect to active external rotation, all patients showed this superior activity of the transferred LD, with an increase of 58% (*p* = 0.002) of the median values of muscular activation. In combined abduction-elevation, this superior muscular activity was present in 11 of the 12 patients, with a median increase of 18% (*p* = 0.009) (Figure 6).

Regarding complications, there was one case of infection that was resolved with a surgical washout and antibiotics that did not influence the final results of the surgery. There were neither neurological nor vascular complications.

## Discussion

The present study has shown that LDT for treatment of irreparable posterosuperior cuff tears relieves pain and improves shoulder function, even in the presence of shoulder pseudoparalysis or failure of previous attempt of cuff repair. Overall, our patients had a significant gain of 26 points in the Constant score, from 33 preoperatively to 59 postoperatively; pain score increased significantly from 7 to 12 points and range of active motion improved a mean of 48° of flexion, 45° of abduction, and 16° of external rotation. Comparable results have been reported by other studies regarding pain, function, and range of motion. The high degree of patient satisfaction was evident, as 78% of our patients would be willing to undergo surgery again.

LDT was introduced by Gerber et al. in 1988 and has since been performed by other authors [8-12,15]. The procedure has proven to be a valid surgical treatment for irreparable posterosuperior cuff tear associated with functional disability and chronic pain. In 1996, Aoki et al. [8] described a series of 12 patients. Good to excellent results were seen in 8 patients at 36 months, with a 36° improvement in elevation of the arm and improved electromyographic activity in 9 of the 12 patients when performing external rotation and abduction. The largest series, described by Gerber et al. in 2006 [16], included 67 patients in which the average Constant score increased from 46 points preoperatively up to 60 points postoperatively. The mean follow-up period was 53 months, and the patients demonstrated significant improvement in spatial control of the arm.

According to most authors, the essential requirements to perform this operation include preservation of subscapularis and deltoid function, acceptable passive shoulder mobility, and the absence of glenohumeral degenerative osteoarthritic disease [8,9,16-19]. In this study, the only case with a poor outcome had a complete tear of the subscapularis muscle. The attempted arthroscopic repair performed in the initial phase of the surgery was not successful in restoring its function of active internal rotation, as detected in the postoperative exam. The biomechanical study of the subscapularis muscle in LDT conducted by Werner et al. [18] in a cadaveric model has revealed the important stabilizing role of the subscapularis in the different motion patterns of the humeral head. The inferior clinical results of LDT in the presence of subscapularis dysfunction reported by most of authors may be explained by the loss of the centering effect of the humeral head upon abduction and elevation if subscapularis function is deficient. The majority of authors [7,8,16-19] agreed that a complete or irreparable subscapularis tear is a definitive contraindication for a LDT. Costouros et al. [20] have reported that fatty infiltration of the teres minor more than grade 2 of the Goutallier classification is a negative predictive factor of the LDT. We cannot conclude about this particular aspect because none of our patients had significant changes of the teres minor.

Contrary to what has been described by Codsi et al. [21], we found that shoulder pseudoparalysis is not a contraindication for this technique, provided that the subscapularis is functional, as demonstrated by the favorable results in three of the four cases in our series. We believe that pseudoparalysis does not guarantee a poor outcome, and that further studies should be performed in this particular subgroup of patients.

LDT proved to be a good treatment option after a failed repair of a massive cuff tear [9,22]. Birmingham and Neviaser [10] reported significant pain relief and improvement in shoulder function in a case series. In our study, all five patients with previous surgery had improved functioning and were satisfied with the results.

Considering the different phases of the LDT, particularly the initial dissection of the LD tendon and the final reinsertion in the humerus, there is no consensus on the choice of surgical technique. Different approach methods have been described, such as dual-incision [12,15] versus single-incision [9,19] as well as the combination with arthroscopic assistance in the reinsertion of the tendon transfer [23-25].

Gerber et al. [7,16] used a double incision with a posterior incision for the detachment of the LD, and a transdeltoid incision to reattach the tendon. Moursy et al. [11] modified the LD detachment by accompanying it with a partial insertion to the humerus bone to obtain a better transosseous fixation of the transplant, reporting better clinical results and tendon integrity.

In order to preserve the deltoid muscle and the coracohumeral arch, Habermeyer et al. [9] described in 2006 a posterior single-incision technique for both steps of the surgery, and Gervasi et al. [23] firstly introduced in 2007 the arthroscopic assistance for the LDT, advocating mini-incision surgery for the withdrawal of the LD tendon, and an arthroscopic fixation of the transfered tendon.

Apart from avoiding a second transdeltoid approach, arthroscopy aids this technique in the crucial part of the LD reinsertion in the greater tuberosity. In our cases, arthroscopy has facilitated the correct placement of the suture anchors and also, depending on the ease of access to the greater tuberosity through the posterior approach, the final tendon attachment can be done either directly or by arthroscopic technique. Recent reports confirm the advantages of the arthroscopic assistance for LDT [24], and other fixation methods have been described such as interference screw or endobutton [25] attempting to strengthen the fixation of the tendon transfer.

Considering the zone of the tendon reinsertion, we followed the directions of Herzberg et al. [14], who described the improved biomechanical behavior of the transplant with reinsertion on the posterosuperior area of the greater tuberosity at the level of the insertion of the infraspinatus. These results were also confirmed by Ling et al. [26]. We used this position and had only one late detachment of the tendon.

Different electromyographic studies have shown that the LD transferred tendon was active postoperatively [8,9,12,13]. Our records demonstrate that the LD transfer is able to adapt functionally to its new anatomical situation, demonstrating increased muscular activation with both external rotation and abduction-elevation movements. Thus, we believe that the LD can provide active external rotation and may act as a depressor with a centering effect on the humeral head, allowing the deltoid to lift and abduct the arm.

As reported in the literature, our data also confirm that the LDT is a surgical procedure with few significant complications [8,9,16].

Several weaknesses exist in the current study. First, it is a small cohort of patients. A larger number of patients would provide more significant information about the safety and efficacy of the procedure, in particular, in the subgroup of patients with pseudoparalysis. A second limitation is that our study cohort was not compared with an alternative control group.

## Conclusions

Our study shows that LDT using a combined open and arthroscopic technique is a valid treatment for irreparable posterosuperior rotator cuff tear, with the requirement that the subscapularis be functional. Additionally, LDT provides a significant improvement in pain and function of the shoulder which endures over time, with a low complication rate. Surface electromyographic examination confirms the increased activation of the transferred LD when performing active movements of external rotation and abduction-elevation. Finally, the combined open and arthroscopic technique is beneficial in the preservation of the deltoid muscle while controlling the correct placement of the suture anchors and helping in the LD reinsertion.

## Additional files

Additional file 1:
**Surface electromyography of latissimus dorsi transfer.** The file describes the surface electromyographic study of latissimus dorsi transfer and the position of electrodes. Additional file 2:
**Latissimus dorsi transfer surgical technique.** The file describes the consecutive steps of the latissimus dorsi surgical technique.

## References

[1] Cofield RH, Parvizi J, Hoffmeyer PH, Lanzer WL, Ilstrup DM, Rowland CM (2001). Surgical repair of chronic rotator cuff tears. A prospective long term study. J Bone Joint Surg Am.

[2] Apoil A, Augereau B (1985). Reparation par lambeau deltoidien des grandes pertes de substance de la coiffe des rotateurs de l´épaule. Chirurgie.

[3] Gartsman GM (1995). Arthroscopic treatment of rotator cuff disease. J Shoulder Elbow Surg.

[4] Walch G, Madonia G, Pozzi I, Riand N, Levigne C, Gazielly D, Gleyze P, Thomas T (1997). Arthroscopic tenotomy of the tendon of the long head of the biceps in rotator cuff ruptures. The cuff. The Cuff.

[5] Fenlin JM, Chase JM, Ruhston SA, Friedman BG (2002). Tuberoplasty: creation of an acromiohumeral articulation—a treatment option for massive, irreparable rotator cuff tears. J Shoulder Elbow Surg.

[6] Boileau P, Chuinard C, Rousanne Y, Bicknell RT, Rochet N, Trojani C (2008). Reverse shoulder arthroplasty combined with a modified latissimus dorsi and teres major tendon transfer for shoulder pseudoparalysis associated with dropping arm. Clin Orthop Relat Res.

[7] Gerber C, Vinh TS, Hertel R, Hess CW (1988). Latissimus dorsi transfer for the treatment of massive tears of the rotator cuff: a preliminary report. Clin Orthop Relat Res.

[8] Aoki M, Okamura K, Fukoshima S, Takahashi T, Ogino T (1996). Transfer of latissimus dorsi for irreparable rotator-cuff tears. J Bone Joint Surg [Br].

[9] Habermeyer P, Magosch P, Rudolph T, Lichtenberg S, Liem D (2006). Transfer of the tendon of latissimus dorsi for the treatment of massive tears of the rotator cuff: a new single-incision technique. J Bone Joint Surg [Br].

[10] Birmingham PM, Neviaser RJ (2008). Outcome of latissimus dorsi transfer as a salvage procedure for failed rotator cuff repair with loss of elevation. J Shoulder Elbow Surg.

[11] Moursy M, Forstner R, Koller H, Resch H, Tauber M (2009). Latissimus dorsi tendon transfer for irreparable rotator cuff tear: a modified technique to improve tendon transfer integrity. J Bone Joint Surg Am.

[12] Henseler JF, Nagels J, Nelissen RG, de Groot JH (2014). Does the latissimus dorsi tendon transfer for massive rotator cuff tears remain active postoperatively and restore active external rotation?. J Shoulder Elbow Surg.

[13] Irlenbusch U, Bernsdorf M, Born S, Gansen HK, Lorenz U (2008). Electromyographic analysis of muscle function after latissimus dorsi transfer. J Shoulder Elbow Surg.

[14] Herzberg G, Urien JP, Dimnet J (1999). Potential excursion and relative tension of muscles in the shoulder girdle: relevance to tendon transfer. J Shoulder Elbow Surg.

[15] Zafra M, Carpintero P, Carrasco C (2009). Latissimus dorsi transfer for the treatment of massive tears of the rotator cuff. Int Orthop.

[16] Gerber C, Maquieira G, Espinosa N (2006). Latissimus dorsi transfer for the treatment of irreparable rotator cuff tears. J Bone Joint Surg Am.

[17] Weening AA, Willems WJ (2010). Latissimus dorsi transfer for treatment of irreparable rotator cuff tears. Int Orthop.

[18] Werner CM, Zingg PO, Lie D, Jacob HA, Gerber C (2006). The biomechanical role of the subscapularis in latissimus dorsi transfer for the treatment of irreparable rotator cuff tears. J Shoulder Elbow Surg.

[19] Lehmann LJ, Mauerman E, Strube T, Laibacher K, Scharf HP (2010). Modified minimally invasive latissimus dorsi transfer in the treatment of massive rotator cuff tears: a two-year follow-up of 26 consecutive patients. Int Orthop.

[20] Costouros JG, Espinosa N, Schmid MR, Gerber C (2007). Teres minor integrity predicts outcome of latissimus dorsi tendon transfer for irreparable rotator cuff tears. J Shoulder Elbow Surg.

[21] Codsi M, Henningam S, Herzog R, Kella S, Kelly M, Leggin B, Williams GR, Iannotti JP (2007). Latissimus dorsi tendon transfer for irreparable posterosuperior rotator cuff tears: surgical technique. J Bone Joint Surg Am.

[22] Pearsall AW, Madanagopal SG, Karas SG (2007). Transfer of the latissimus dorsi as a salvage procedure for failed debridement and attempted repair of massive rotator cuff tears. Orthopedics.

[23] Gervasi E, Causero A, Parodi PC, Raimondo D, Tancredi G (2007). Arthroscopic latissimus dorsi transfer. Arthroscopy.

[24] De Cupis V, De Cupis M (2012). Massive cuff tears treated with arthroscopically assisted latissimus dorsi transfer. Surgical technique. Muscles Ligaments Tendons J.

[25] Goldstein Y, Grimberg J, Valenti P, Chechik O, Drexler M, Kany J (2013). Arthroscopic fixation with a minimally invasive axillary approach for latissimus dorsi transfer using an endobutton in massive and irreparable postero-superior cuff tears. Int J Shoulder Surg.

[26] Ling HY, Angeles JG, Horodyski MB (2009). Biomechanics of latissimus dorsi transfer for irreparable posterosuperior rotator cuff tears. Clin Biomech.

